# The effect of acupoint herbal patching on the quality of life of patients recovering from COVID-19: A protocol for systematic review and meta analysis

**DOI:** 10.1097/MD.0000000000025979

**Published:** 2021-05-14

**Authors:** Heran Wang, Hailin Jiang, Jinying Zhao, Xuewei Zhao, Yiran Han, Meng Meng, Ting Pan, Tie Li, Fuchun Wang

**Affiliations:** a School of Acupuncture-Moxibustion and Tuina; b Graduate school, Changchun University of Chinese Medicine; c Department of Acupuncture, The Affiliated Hospital of Changchun University of Chinese Medicine, Changchun, China.

**Keywords:** acupoint herbal patching, COVID-19, protocol, systematic

## Abstract

**Background:**

There is a worldwide outbreak of COVID-19, and as the number of patients increases, an increasing number of patients are recovering. However, no relevant systematic review or meta-analysis has been designed to evaluate the effects of acupoint herbal patching on the life of patients recovering from COVID-19.

**Methods:**

The following electronic databases will be searched from the respective dates of database inception to April 20, 2021: The Cochrane Library, Web of Science, EMBASE, MEDLINE, China National Knowledge Infrastructure (CNKI), Chinese Biomedical Literature Database (CBM), Wanfang database, the Chinese Scientific Journal Database (VIP), and other sources. All published randomized controlled trials in English or Chinese related to acupoint herbal patching for COVID-19 will be included. The primary outcome was the timing of the influence of acupoint herbal patching on the quality of life of convalescent patients. Secondary outcomes were accompanying symptoms (such as myalgia, expectoration, stuffiness, runny nose, pharyngalgia, anhelation, chest distress, dyspnea, crackles, headache, nausea, vomiting, anorexia, diarrhea) disappearance rate, negative COVID-19 results rate on two consecutive occasions (not on the same day), average hospitalization time, clinical curative effect, and improved quality of life.

**Results:**

The main purpose of this systematic review protocol was to assess the effectiveness and safety of acupoint herbal patching therapy for treating patients recovering from COVID-19.

**Conclusion:**

The conclusion of our study will provide evidence to judge whether acupoint herbal patching is an effective intervention for the quality of life in patients recovering.

**PROSPERO registration number:**

CRD42021246550

## 1 Introduction

Coronavirus disease (COVID-19), the infection caused by severe acute respiratory syndrome coronavirus 2 (SARS-CoV-2), was first reported on December 31, 2019.^[1]^ This virus has spread rapidly across mainland China and worldwide. On March 11, the World Health Organization declared a pandemic state worldwide.^[2–5]^ COVID-19 patients show typical respiratory symptoms, such as cough, fever, and lung damage, and other symptoms such as fatigue, myalgia, and diarrhea.^[6]^ The studies indicate that the main clinical manifestations of COVID-19 are fever (90% or more), cough (approximately 75%), and dyspnea (up to 50%). A small, but significant subset of patients has gastrointestinal symptoms. Preliminary estimates of case fatality, likely to fall as better early diagnostic efforts come into play, is approximately 2%.^[7]^ To date, there has been no effective drug to reduce infection and pandemic burden.^[8,9]^ The importance of rehabilitation after COVID-19 has been emphasized according to the framework of the International Classification of Functioning, Disability, and Health.^[10]^ The WHO does not have rehabilitation guidelines for patients post–COVID-19.^[11]^ Therefore, a novel treatment strategy can effectively manage the quality of life of patients recovering from COVID-19 without serious adverse events.

Acupoint herbal patching (AHP) is an ancient Chinese medicine method in which acupuncture points on the skin are manually stimulated with herbs. AHP is a treatment method to prevent diseases by strengthening the immune system through the stimulation of acupuncture points with a small amount of various herbal preparations.^[12,13]^ The earliest records of AHP are traced back to the classic Prescriptions for Fifty-two Diseases (Wu Shi Er Bing Fang), where AHP was listed as a treatment method, and it is still widely used today.^[14–16]^ AHP is a herbal patch that is applied to specific acupoints to stimulate the skin, meridians, and collaterals to produce preventive and therapeutic effects.^[17]^ Statistics from China suggest that approximately 100,000 people are treated with AHP every summer, and this number has increased by 13.14% from 2013 to 2014. At present, acupoint herbal patching as an adjuvant therapy is currently undergoing clinical trials in the Chinese Acupuncture Society, and the China Academy of Chinese Medical Sciences has selected more than 10 hospitals.^[18]^ Acupoint herbal patching has the advantages of rapid action and fewer side effects, simple and convenient, short course of treatment, good efficacy, safety and reliability, and easy acceptance by patients.

Therefore, exploring more effective and safe treatments and improving the prognosis of patients with COVID-19 has become an important health problem. There is an urgent need to improve the quality of life of convalescent patients.^[19]^ It is necessary to conduct a systematic review to provide a convincing conclusion as to whether acupoint herbal patching is an appropriate method to treat COVID-19.

## 2 Methods

### 2.1 Design and registration of the review

This systematic review protocol was registered with PROSPERO (registration number: CRD42021246550). We followed the Preferred Reporting Items for Systematic Reviews and Meta-analysis Protocol (PRISMA-P)^[20]^ to accomplish the systematic review protocol. This study was conducted for the secondary collection and analysis of original RCT data, so ethical approval or patient informed consent is not required.

### 2.2 Inclusion criteria for study selection

#### 2.2.1 Type of study

We will include articles related to acupoint herbal patching therapy for patients recovering from COVID-19. Due to language restrictions, we will search for articles in English and Chinese in order to obtain a more objective and accurate evaluation, and RCTs of acupoint herbal patching therapy for patients recovering from COVID19 will be eligible for inclusion.

#### 2.2.2 Types of participants

All patients recovering from COVID-19 will be included regardless of sex, age, race, education, and economic status. Postoperative infections, psychopaths, and severe dermatosis were not included.

#### 2.2.3 Types of interventions and comparisons

Participants in the intervention group were those who underwent acupoint herbal patching, regardless of the herbal regimen, acupoints selected, and patching time. There will be no restrictions on the age and original countries of the participants. In the control group, patients received medication, no treatment, sham or placebo acupoint catgut embedding, acupuncture/electro-acupuncture, etc. In addition, a review of trials evaluating acupuncture combined with another treatment compared with other typical treatments alone will be included.

#### 2.2.4 Types of outcome measures

The primary outcome was the influence of acupoint herbal patching on the quality of life of convalescent patients, and the secondary outcomes included accompanying symptoms (such as myalgia, expectoration, stuffiness, runny nose, pharyngalgia, anhelation, chest distress, dyspnea, crackles, headache, nausea, vomiting, anorexia, diarrhea) disappearance rate, negative COVID-19 results rate on 2 consecutive occasions (not on the same day), CT image improvement, average hospitalization time, occurrence rate of common type to severe form, clinical cure rate, and mortality.

### 2.3 Data sources

The main sources of information that will be obtained in this study include electronic resource databases, which will be searched from the respective dates of database inception to April 21, 2021. We plan to search eight English and Chinese electronic databases, including the Web of Science, Cochrane Library, PubMed, EMBASE, SinoMed, Wanfang, China Science and Technology Journal (VIP), and China National Knowledge Infrastructure (CNKI) databases, for potentially eligible studies. We will also search for dissertations, conference proceedings, and reference lists of relevant included studies.

### 2.4 Search strategy

**Figure 1:**
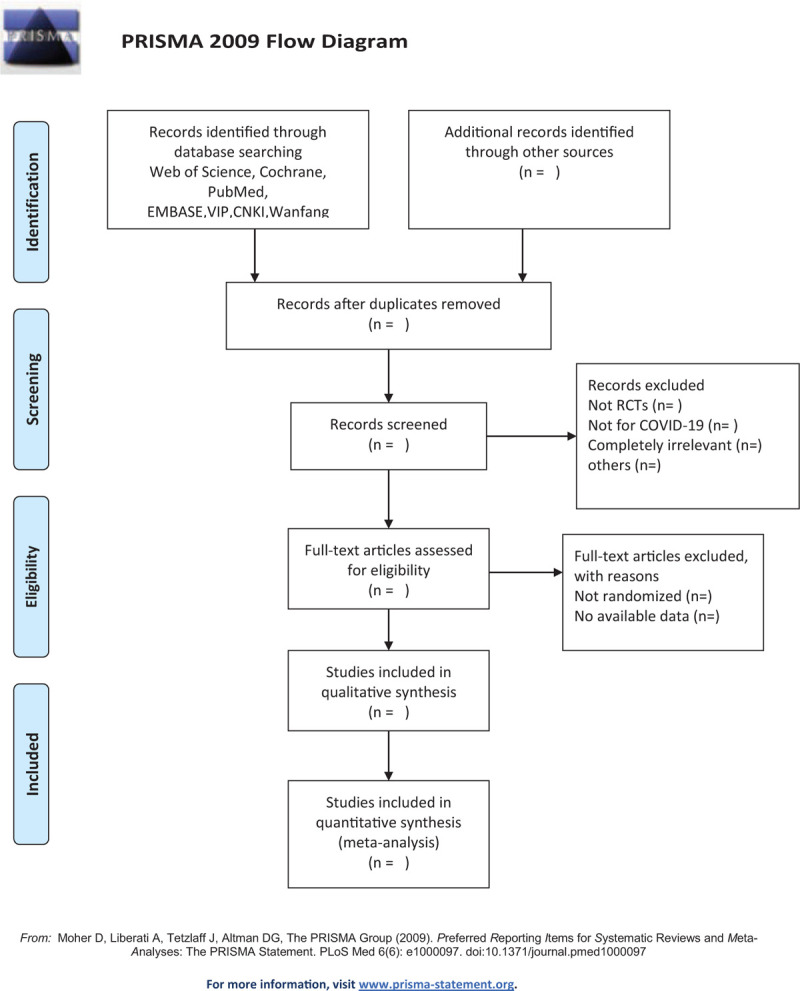
The PRISMA flow chart of selection process.

The strategy was created according to the Cochrane Handbook guidelines. All published RCTs on this subject were included. The primary selection process is shown in the PRISMA flowchart (Fig. 1). The exemplary search strategy of WOS is listed in Table 1, and search terms conform to the medical subject heading. According to the different retrieval modes, keywords may be combined with free words, and an appropriate search mode will be performed. The following search keywords will be used: (“acupoint application” or “acupoint sticker” or “crude herb moxibustion” or “medicinal vesiculation” or “herbal patch” or “herbal plaster” or “acupoint patch” or “acupoint sticking” or “point application therapy” or “drug acupoint application” or “acupuncture point application therapies” or “plaster therapy” or “external application therapy” or “acupoint herbal patching”) and (“COVID-19”or”Corona Virus Disease 2019” or “Corona Virus”) and); (“convalescence” or “rehabilitation” or “convalescent period” or “decubation”); (“randomized controlled trial” or “controlled clinical trial” or “random allocation” or “randomized” or “randomly” or “double-blind method” or “single-blind method” or “clinical trial”). Similar but adaptive search strategies can be applied to other electronic databases. Language is restricted to English and Chinese. The details of the PubMed database search strategies are presented in Table 1.

**Table 1:** Web of science search strategy.

Number	Terms
#1	acupoint herbal patching
#2	acupoint sticker
#3	crude herb moxibustion
#4	medicinal vesiculation
#5	herbal patch
#6	herbal plaster
#7	acupoint patch
#8	acupuncture point application therapies
#9	acupoint sticking
#10	acupuncture point
#11	point application therapy
#12	drug acupoint application
#13	plaster therapy
#14	external application therapy
#15	acupoint application
#16	#1 or #2–15
#17	COVID-19
#18	Corona Virus Disease 2019
#19	Corona virus
#20	#17 or #18 to 19
#21	Rehabilitation
#22	Convalescence
#23	convalescent period
#24	Decubation
#25	#21 or #22–24
#26	randomized controlled trial
#27	controlled clinical trial
#28	random allocation
#29	Randomized
#30	Randomly
#31	double-blind method
#32	single-blind method
#33	clinical trial
#34	#26 or #27-33

### 2.5 Data collection and analysis

#### 2.5.1 Selection of studies

Two authors will independently select clinical trials conforming to the inclusion criteria. After the articles were screened, disrelated, repetitive, nonstandard literature was excluded. We will also try to obtain the full text, and the obtained literature will be managed using EndNote software, V.X8 (United States). The selection process is shown in the PRISMA flow chart (http://www.prismastatementorg/) (Fig. 1). If the full literature is unable to obtain or related data is incomplete, we will contact the corresponding author. In case of disagreement between the two reviewers, third-party experts will be consulted to determine the selection divergence.

#### 2.5.2 Data extraction and management

Two independent authors collected data from the selected eligible articles entered into an Excel form. The extracted information included the reference ID, name of the first author, time of publication, country, participant characteristics (height, weight, sex), intervention, randomization, study characteristics (press, nationality, journals, research design), outcome measures, duration of follow-up, adverse effects, and other detailed information. If there is any disagreement between the two authors in the literature data extraction, a third article participant will discuss the decision together. If necessary, we will contact the corresponding authors of the trials as much as possible for further information.

#### 2.5.3 Assessment of the risk of bias and reporting of study quality

Two review authors independently evaluated the risk of bias to evaluate the quality of the studies using the Cochrane Collaboration's risk-of-bias assessment method. The following domains will be evaluated: random sequence generation, blindness of participants and staff, attrition bias, detection bias, selective result reporting, and other sources of bias. The risk of bias will be assessed and classified according to three levels: low risk, unclear risk, and high risk. Any discrepancies were resolved through discussions and negotiations with the third author. When a consensus on risk assessment cannot be reached by discussion, the third reviewer will make the decision.

#### 2.5.4 Measures of treatment effect

Two authors will independently and cross-check the treatment effect by Review Manager 5.3.5, provided by the Cochrane Collaboration. Risk ratios (RRs) with 95% confidence intervals (CIs) were adopted if dichotomous data existed. Continuous data will be presented as the mean difference or standard mean difference with a 95% CI. Other binary data were converted into the RR form for analysis.

#### 2.5.5 Management of missing data

We will try our best to ensure data integrity. If the necessary data in the literature may be lacking, we will contact the corresponding authors by email or other contacts. If the missing data are unavailable, an intent-to-treat analysis will be performed as much as possible (the analysis should include data from all participants in the initially randomly assigned group), and a sensitivity analysis will be performed to determine if the results are inconsistent.

#### 2.5.6 Assessment of heterogeneity

Cochrane Handbook for Systematic Reviews of Interventions^[21]^ The heterogeneity of studies will be evaluated by the χ^22^ test will be used to detect statistical heterogeneity and the I^2^ statistic will be used to quantify inconsistency with RevMan5.3.5. The following criteria will be used: when the I^2^ test value is < 50% and *P* value > 1, we think there is no heterogeneity between these trials, and when the I^2^ test value is > 50% and the *P* value is < 1, there is significant heterogeneity between the included trials. The random-effects model will be applied if heterogeneity is still important.

#### 2.5.7 Assessment of reporting bias

Funnel plots were created to assess reporting bias; once > 10 trials were included, funnel plots could be used to test for reporting bias. Dissymmetry funnel plots indicate a high risk of reporting bias, while symmetric funnel plots indicate low risk.

#### 2.5.8 Data synthesis

RevManV.5.3.5. software was used for all statistical analyses. We decided to use either a fixed-effects or random-effects model based on the heterogeneity levels of the included studies. If no substantial statistical heterogeneity is detected, the data synthesis will be processed using the fixed-effects model, and if substantial statistical heterogeneity is detected, the data synthesis will be performed using the random-effects model.

If a significant level of heterogeneity is found, a descriptive analysis will be performed.

#### 2.5.9 Subgroup analysis

Subgroup analysis was performed based on the findings of the data synthesis. Factors such as different types of control interventions and different outcomes will be considered, and subgroup analysis will be conducted relevant to these categories.

#### 2.5.10 Sensitivity analysis

We will conduct a sensitivity analysis to identify whether the conclusions are robust in the review according to the following criteria: sample size, heterogeneity qualities, and statistical model (random-effects or fixed-effects model).

#### 2.5.11 Grading the quality of evidence

The Grade of Recommendations Assessment, Development and Evaluation (GRADE) will be a tool to evaluate the quality of the evidence,^[22]^ and will rate the quality by the following levels: very low, low, moderate, or high 4 levels.

## 3 Discussion

To the best of our knowledge, there is no planned or published systematic review of the effectiveness and safety of AHP for patients recovering from COVID-19. The purpose of this study was to evaluate the effect of AHP on the quality of life of convalescent patients, accompanying symptom disappearance rate, negative COVID-19 results rate on two consecutive occasions, average hospitalization time, and clinical curative effect.^[23,24]^ This study is the first to evaluate the clinical efficacy and effective prescription of AHP for patients recovering from COVID-19 and may benefit practitioners in the field of complementary and alternative therapies.

## 4 Ethics and dissemination

Ethical approval or patient informed consent was not required because this work was carried out using published data. We aimed to explore the clinical efficacy rate, functional outcomes, quality of life, and improvement of clinical symptoms of gastric ulcers. Finally, the results will be submitted to a peer-reviewed journal.

## Author contributions

**Conceptualization:** Heran Wang, Hailin Jiang, Yiran Han.

**Data curation:** Jinying Zhao, Meng Meng.

**Formal analysis:** Heran Wang, Jinying Zhao.

**Funding acquisition:** Fuchun Wang.

**Investigation:** Xuewei Zhao, Ting Pan.

**Methodology:** Jinying Zhao, Meng Meng.

**Project administration:** Tie Li, Fuchun Wang.

**Supervision:** Fuchun Wang.

**Validation:** Heran Wang, Yiran Han.

**Visualization:** Hailin Jiang, Yiran Han.

**Writing – original draft:** Heran Wang, Hailin Jiang.

**Writing – review & editing:** Heran Wang, Tie Li, Fuchun Wang.
